# Early Assessment of Colorectal Cancer by Quantifying Circulating Tumor Cells in Peripheral Blood: ECT2 in Diagnosis of Colorectal Cancer

**DOI:** 10.3390/ijms18040743

**Published:** 2017-03-31

**Authors:** Chih-Jung Chen, Wen-Wei Sung, Hung-Chang Chen, Yi-Jye Chern, Hui-Ting Hsu, Yueh-Min Lin, Shu-Hui Lin, Konan Peck, Kun-Tu Yeh

**Affiliations:** 1 Department of Surgical Pathology, Changhua Christian Hospital, Changhua 8864, Taiwan; 132540@cch.org.tw (C.-J.C.); javawomanfanny@gmail.com (H.-T.H.); 93668@cch.org.tw (Y.-M.L.); 74630@cch.org.tw (S.-H.L.); 2 Department of Medical Technology, Jen-Teh Junior College of Medicine, Nursing and Management, Miaoli 88637, Taiwan; flutewayne@gmail.com; 3 School of Medicine, Chung Shan Medical University, Taichung 8864, Taiwan; 4 Department of Urology, Chung Shan Medical University Hospital, Taichung 8864, Taiwan; 5 Institute of Medicine, Chung Shan Medical University, Taichung 8864, Taiwan; 6 Department of Medical Education, Chung Shan Medical University Hospital, Taichung 8864, Taiwan; 7 Department of Colon and Rectal Surgery, Changhua Christian Hospital, Changhua 8864, Taiwan; 54464@cch.org.tw; 8 Institute of Biomedical Sciences, Academia Sinica, Taipei 8862, Taiwan; yijyechern@gmail.com; 9 Institute of Medicine, Chung Shan Medical University, Taichuang 8864, Taiwan

**Keywords:** circulating tumor cell, ECT2, CEA, colorectal cancer, biomarker

## Abstract

Circulating tumor cells (CTCs) in peripheral blood is an indication of poor prognosis for patients with different cancer types. However, most of the available technologies for detecting CTCs show low sensitivity and specificity. Therefore, we attempted to find an alternative marker for CTCs of colorectal cancer. We have directly extracted RNA from CTCs contained in 1.5 mL peripheral blood from 90 colorectal cancer patients and 151 healthy donors, and screened these samples for candidate marker genes by nested real-time quantitative polymerase chain reaction (PCR). From genes selected from a public database of microarray analyses, we successfully identified epithelial cell transforming sequence 2 oncogene (*ECT2*) as a gene that exhibits high differential expression ratios (*p* < 0.01). ECT2 displays good sensitivity and specificity, with an area under the curve (AUC) value of 0.821. This marker gene also has a high detection rate in patients with serum carcinoembryonic antigen (CEA) concentrations below the diagnostic threshold of 5 ng/mL. The expression of ECT2 can therefore serve as an alternative measurement that can compensate for the inadequacy of the current CEA test in the diagnosis and monitoring of colorectal cancer patients.

## 1. Introduction

The clinical outcomes and treatment of patients with colorectal cancer vary depending on tumor location and stage at diagnosis [1]. Patients whose colorectal cancers are detected at a localized stage have a significantly higher 5-year relative survival rate when compared with patients whose cancers have spread regionally to involve adjacent organs or lymph nodes (5-year relative survival rate: 90.1% vs. 69.2%). Notably, when the tumors have spread to distant organs, the 5-year survival rate is only 11.7% [2,3].

For colorectal cancer patients diagnosed in early stages, surgery to remove the tumor and the nearby lymph nodes is the most common treatment [3,4]. In contrast, patients with late-stage disease often undergo chemotherapy, alone or in combination with radiation therapy, before or after surgery [3,4,5]. The efficacy of current cancer therapy is currently assessed based on imaging methods that measure the size of the tumor mass before and after therapy. This assessment is usually done several weeks after the therapy so that an appreciable reduction in tumor size can be observed; however, metastasis might also occur during this time interval. In addition, a large proportion of early-stage patients still develop distant metastasis even though they undergo surgical removal of the tumor mass [6]. Consequently, a reliable method to detect cancer cells prior to metastatic tumor formation could improve the treatment decisions for colorectal cancer patients.

Cancer-related death is most commonly caused by cancer metastasis driven by the epithelial to mesenchymal transition (EMT) process [7,8]. Successful metastasis requires that the cancer cells have the capability to enter the blood circulation, attach to the endothelium, invade the target distant organs, and subsequently form tumors [7,9]. The presence of circulating tumor cells (CTCs) in the blood stream indicates that cancer cells have undergone the first step and have entered the blood circulation [9,10,11,12]. CTCs can be shed into the mesenteric circulation or lymph vessels and initiate overt metastases via these routes [8,9,10,12,13]. Therefore, a successful method for detection of CTCs in the blood circulation could substantially improve the diagnosis and treatment of colorectal cancer patients prior to full blown metastasis.

The detection of CTCs associated with colorectal cancer has been attempted by measuring the specific mRNA expression of several marker genes in blood samples [14,15,16]. Among those genes, *carcinoembryonic antigen* (*CEA*) was identified as a possible marker to predict cancer relapse, clinical outcome, and tumor malignancy [17,18,19]. The disruption of normal tissue architecture in malignancy and the loss of polarization of neoplastic cells located deep inside the tumor glandular tissue results in the expression of CEA over the entire cell surface. Shedding of these cells into the bloodstream eventually leads to a rise in serum CEA levels [17,18,19]. However, CEA alone is not useful as a diagnostic marker and the mean rate of detection of CTCs by CEA measurement is less than 40% [20,21]. Thus, new markers or combinations of markers are needed to improve the detection of CTCs in serum from cancer patients.

The aim of the present study was to establish a rapid quantitative polymerase chain reaction (qPCR) method for evaluating therapy efficacy based on quantifying the numbers of CTCs in peripheral blood. Candidate genes were screened from a public database of microarray analyses of mRNA expression. The convenience of rapid blood taking makes this assessment method useful for regular monitoring of tumor growth and the effectiveness of chemotherapy drugs.

## 2. Results

### 2.1. Identification of ECT2 as a Candidate Marker Gene for Quantifying Circulating Tumor Cells in Colorectal Cancer Patients

**Figure 1:**
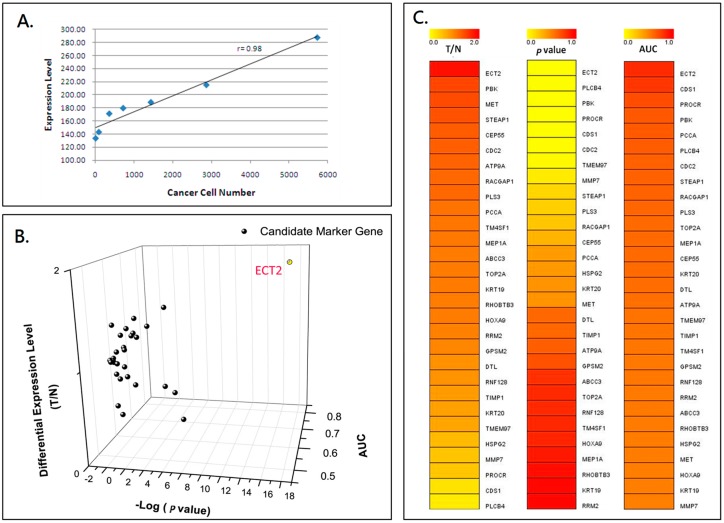
mRNA expression of candidate marker genes of peripheral blood cells for detecting circulating tumor cells in colorectal cancer patients. (**A**) This scatter plot shows that the relative expression of the tested gene is linearly correlated with cancer cell numbers. The curve was generated by taking the mean of triplicates of each data point. (**B**) The 3D scatter plot shows the distribution of three evaluation criteria: *p*-value, area under the curve (AUC), and gene expression ratio (Patients vs. Normal) for 29 tested marker genes. The epithelial cell transforming sequence 2 oncogene (*ECT2*) gene had the highest differential gene expression ratio, largest area under the curve, and the most significant *p*-value among all tested genes. (**C**) The three heatmaps show the individual ranking of each tested marker gene for the three evaluation criteria.

The clinicopathological characteristics of the study subjects are listed in Table 1. Total RNA extracted from the two groups was used for further screening of candidate marker genes by nested real-time qPCR according to the flowchart shown in Figure S1. The results of gene expression according to the microarray analyses from the public database were collected to construct the list of potential CTC detection markers. The candidate marker genes for detecting CTCs were further evaluated by analysis of the mRNA expression in the two groups, as shown in Figure 1. The detection method was calibrated by semiquantitative estimation of the number of CTCs in the peripheral blood from the cancer patients. The differential expression ratio (epithelial cell transforming sequence 2 oncogene—ECT2/ Glyceraldehyde 3-phosphate dehydrogenase—GAPDH) of ECT2 and the number of cancer cells was linearly correlated, as shown by the scatter plot used to confirm the RNA extraction quality (*r* = 0.97, Figure 1A). The candidate genes were selected from the 3D scatter plot distribution of 29 marker genes using three evaluation criteria: *p*-value, area under curve (AUC), and gene expression ratio (T/N: Patients vs. Normal) (Figure 1B). The individual ranks of each tested marker gene for the three criteria are shown in three heatmaps (Figure 1C). The 3D scatter plots and the heatmaps identified ECT2 as having the highest differential gene expression ratio (T/N = 1.77), the largest area under curve (AUC = 0.821), and the most significant *p*-value (*p* < 0.001) among all tested genes, including PDZ binding kinase (PBK) and MET proto-oncogene, receptor tyrosine kinase (MET).

**Figure 2:**
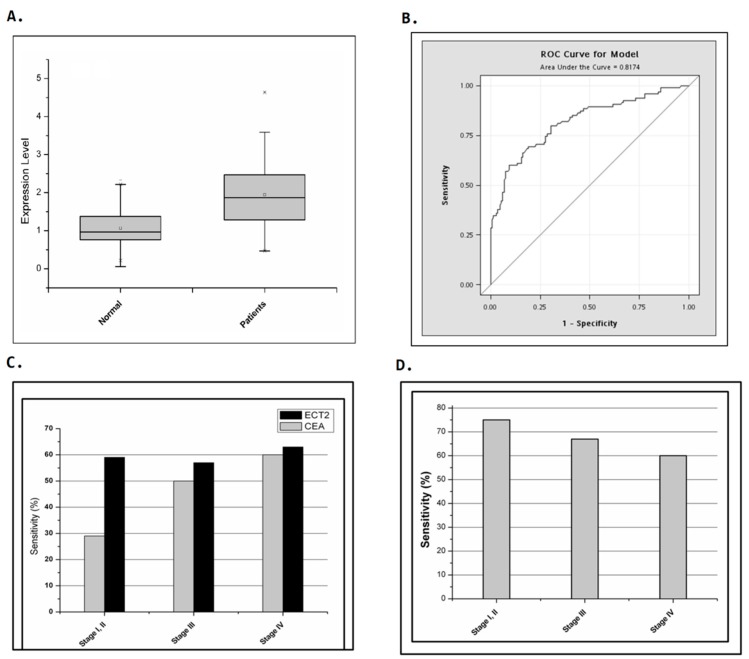
Identification of *ECT2* as a candidate marker gene for quantifying circulating tumor cells in colorectal cancer patients. Comparison of the detection sensitivity of CEA and *ECT2*, singly and in combination, as markers in colorectal cancer patients at different stages. (**A**) A box plot showing differential expression ratios of *ECT2* in colorectal cancer patients and a normal control group. The *p* value of a two-tailed unpaired *t*-test was 1.42 × 10^−15^. The whiskers of the boxes indicate 1.5 interquartile range (IQR) of the lower and higher quartile. (**B**) Receiver operating characteristic (ROC) analysis of ECT2 expression in cells from peripheral blood of colorectal cancer patients and normal donors. The area under curve (AUC) was 0.821. (**C**) A total of 90 colorectal cancer patients were included in this study. CEA had a detection sensitivity of 29%, 50%, and 60%, while *ECT2* had a sensitivity of 59%, 57%, and 63%, in patients with stage I/II, stage III, and stage IV cancers, respectively. (**D**) In patients with serum CEA lower than the diagnostic threshold of 5 ng/mL, the ECT2 quantification sensitivity in patients with stage I/ II, stage III, and stage IV were 75%, 67%, and 59%.

Further confirmation of ECT2 as a possible marker for CTC detection was obtained by estimating the mRNA expression of ECT2 by cells from peripheral blood from colorectal patients and non-cancer donors. Significantly higher mRNA expression of ECT2 was detected in the peripheral blood from colorectal cancer patients than from the non-cancer donors (mean of ECT2 expression: 0.97 vs. 1.88, normal vs. patients, *p* = 1.42 × 10^−15^, Figure 2A). The receiver operating characteristic analysis of ECT2 expression in cells from peripheral blood from colorectal cancer patients and normal donors gave an AUC value of 0.817. Therefore, we successfully identified ECT2 as exhibiting a high differential gene expression ratio (*p* < 0.001) and good sensitivity and specificity for CTCs.

### 2.2. Comparison of the Detection Sensitivity of CEA and ECT2 in Colorectal Cancer Patients at Different Stages

Since *CEA* had been suggested as a candidate gene for monitoring surgically curable recurrence in colorectal cancer patients, we compared the sensitivity of CEA and ECT2 in our study group based on tumor stages. In the 90 colorectal cancer patients in this study, the detection sensitivity of CEA increased from 29% to 60% depending on the cancer stage (stage I/II, stage III, and stage IV: 29%, 50%, and 60%, respectively, Figure 2C). However, the detection sensitivity of ECT2 was approximately 60% for all the different stages (stage I/II, stage III, and stage IV: 59%, 57%, and 63%, respectively, Figure 2C). Interestingly, a high detection rate was obtained using ECT2 in patients whose serum concentration of CEA was lower than the diagnostic threshold of 5 ng/mL; this was especially the case in patients with early stage cancer (ECT2 quantification sensitivity in patients with stage I/ II, stage III, and stage IV: 75%, 67%, and 59%, respectively, Figure 2D). The cut-off level for the differential expression ratio of ECT2 was 1.79 for a specificity of 90%. These results indicated that ECT2 had better sensitivity than CEA for detecting CTCs in blood from colorectal patients.

**Figure 3:**
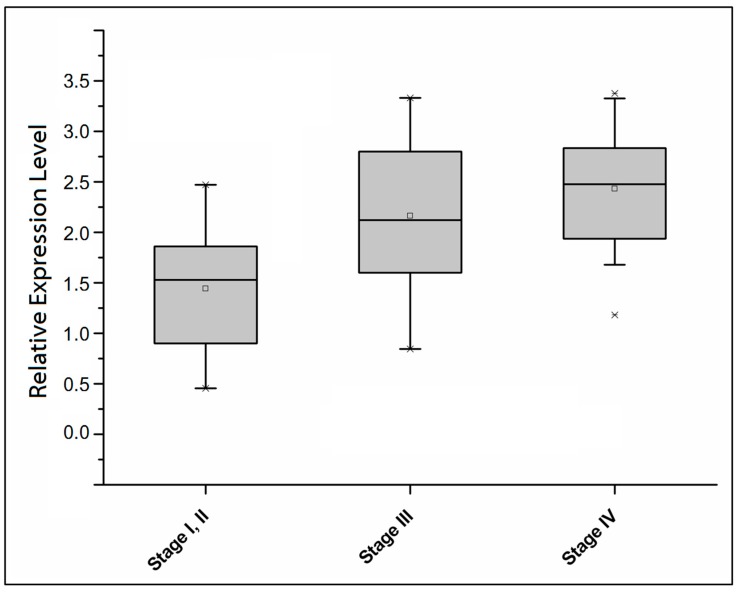
Increasing expression of *ECT2* in colorectal cancer patients with serum CEA levels greater than 5 ng/mL and advancing disease. The box plot shows differential expression ratios of ECT2 in patients with detectable serum CEA levels (more than 5 ng/mL). The *p-*value of a Kruskal-Wallis (KW) test was less than 0.05.

The expression of *ECT2* was increased in advanced stage patients whose serum CEA levels were greater than 5 ng/mL (mean of ECT2 expression level in patients with stage I/II, stage III, and stage IV: 1.53, 2.12, and 2.48, respectively, *p-*value of Kruskal-Wallis (KW) test <0.05, Figure 3). A combination of CEA and ECT2 measurements resulted in detection sensitivity of more than 80% in all patients with different stages (detection sensitivity of patients in stage I/II, stage III, and stage IV: 82%, 87%, and 84%, Table 2). Thus, ECT2 expression can be considered as an alternative measure that can compensate for the inadequacy of the current CEA test for monitoring and prognosis of colorectal cancer patients.

## 3. Discussion

Early detection of the presence of metastatic colorectal cancer cells before metastatic tumor formation might improve treatment decisions and the clinical outcome of patients, especially for patients without visible metastasis. In the present study, we found that detection of ECT2 mRNA expression in peripheral blood cells could be a reliable method for detecting the presence of CTCs. In our study group, better sensitivity was obtained for detection of ECT2 in peripheral blood cells than for detecting serum CEA levels (Figure 2C). Moreover, the higher ECT2 expression in late stage compared to early stage patients with high CEA expression suggested that ECT2 might predict tumor stage and clinical outcome in colorectal patients (Figure 3). However, further study that includes a larger study population should be conducted to confirm the role of ECT2 in predicting the presence of CTCs and poor prognosis in colorectal cancer patients.

The evaluation of CTC count before and during treatment could independently predict clinical outcome of advanced colorectal cancer patients treated with chemotherapy plus targeted agents [8,11,12,19,22,23,24,25,26]. In the present study, CTCs were defined as isolated and nucleated cells that were positive for the expression of epithelial cell adhesion molecule and cytokeratin but negative for cluster of differentiation 45 (CD45) expression [27]. The combined analysis of CTC count and the result of computed tomography imaging provided a more accurate outcome assessment [26]. However, the identification of CTCs by markers is expensive and impractical for screening of all colorectal cancer patients. Thus, a simple and sensitive method to detect CTCs might be useful as a first screen for the possible cancer metastasis [8]. In this study, we focused on the role of ECT2 expression level in CTCs of colorectal cancer patients. Other possible candidates are still under further investigation.

*ECT2*, also known as ARHGEF31, is elevated with the onset of DNA synthesis and remains elevated during the G2 and M phases [28,29,30]. *ECT2* belongs to the Dbl family that possesses a Dbl homology/pleckstrin homology cassette in the COOH terminus and mediates the guanine nucleotide exchange of Rho GTPases [31]. The NH_2_ terminus of ECT2 contains tandem repeats of the BRCA1 C Terminus (BRCT) domain, which is involved in cell cycle regulation and DNA damage responses [32]. In lung cancer and esophagus cancer, cDNA microarrays revealed that ECT2 was frequently overexpressed in the tumors [33]. Tumor tissue microarray analysis that investigated ECT2 expression in tumors and its prognostic value indicated that this gene is a likely prognostic biomarker and a potential therapeutic target for developing anticancer drugs for lung and esophagus cancers [33]. Knock-down of ECT2 expression resulted in inhibition of tumor growth in both lung and esophageal cancer cell lines [33]. The invasiveness of mammalian cells was enhanced by the introduction of ECT2 [33,34,35]. In aggressive clear cell renal cell carcinoma, the ECT2 expression correlated with pituitary tumor-transforming 1 (PTTG1) expression and poor clinical outcomes, suggesting a possible downstream molecular mechanism whereby ECT2 promoted cancer malignancy [34]. A bioinformatic analysis of 34 candidate marker genes of multiple case-matched normal and colorectal tumor tissues identified ECT2 mRNA as a potential colorectal cancer biomarker [36]. Colorectal cancer patients with low tumor/normal expression ratios had more advanced lymphatic invasion than did patients with high expression ratios, which suggested that tumors that express high levels of ECT2 might have lower invasion capability [36]. Overall, abundant studies have confirmed a role for ECT2 in promoting tumor malignancy, but no further evidence has been presented to support a suppressive role of ECT2 in tumor malignancy.

CTCs may be responsible for metastasis, and enormous effort has been made to analyze the clinical impacts of CTCs. Consequently, different strategies and applicable technologies or devices for reliable identification, isolation, and further analysis of CTCs have been proposed [9,13,37]. We previously reported the successful detection and quantification of CTCs from the peripheral blood of lung cancer patients by modification of a method for quantifying mRNA expression from band (integrated) density by hydrolysis of a fluorescent probe. These refinements improved the reliability and repeatability of the test. However, like most studies in this field, prospective multicenter trials should be performed to validate our findings. Nevertheless, our present results revealed that ECT2 in peripheral blood could be a biomarker for predicting CTCs in colorectal cancer patients. The current CEA test shows moderately to significantly elevated levels in different kinds of diseases and is not unique to colorectal cancer. In contrast, the expression of ECT2 was increased in advanced stages and had higher sensitivity as a marker for predicting CTCs when compared to serum CEA [38]. These results suggested that a more intensive follow-up or aggressive treatment for patients showing high ECT2 expression might promote early detection of tumor metastasis in colorectal patients.

## 4. Materials and Methods

### 4.1. Ethics Statement

This study was approved by the institutional review board and ethical committee of Changhua Christian Hospital, Changhua, Taiwan (IRB serial number: 070306) and Academia Sinica, Taipei, Taiwan (IRB serial number: AS-IRB02-07023). The data were analyzed anonymously and written informed consent from the participants was obtained.

### 4.2. Study Subjects

This study included 90 colorectal cancer patients and 151 non-cancer controls. The blood and tumor tissues of colorectal cancer patients were collected at Changhua Christian Hospital. Colorectal cancer specimens were surgically collected from colorectal cancer patients between 2007 and 2008. No patient underwent preoperative radiotherapy, chemotherapy, or any other treatment. Cancers were staged according to the American Joint Committee on Cancer (AJCC) Colon Cancer Staging, 7th edition (2009). The clinical data, including sex, age, TNM stages, and follow-up information including the serum CEA level, were obtained from medical records and the cancer registry.

### 4.3. Sample Collection and RNA Preparation of Circulating Tumor Cells

RNA from tissues and blood was extracted and reverse transcribed for testing the candidate CTC detection markers. Briefly, samples were collected from each subject with Vacutainers (Becton Dickinson, Rutherford, NJ, USA). The first tube with 1 to 2 mL of peripheral blood was discarded and only the second tube with 3 to 4 mL of blood was assayed to avoid epithelial cell contamination by the needle when it pierced through the skin. Total cellular RNA was extracted with the QIAamp RNA Blood Mini kit (Qiagen, Hiden, Germany). The detailed procedures for sample collection were described previously [39,40].

### 4.4. Microarray Analysis and Nested Real-Time Quantitative Polymerase Chain Reactions

Paper searches of gene expression microarray analysis were conducted to construct the list of potential CTC detection markers. Nested real-time quantitative polymerase chain reactions, using TaqMan (Thermo Fisher Scientific, Waltham, MA, USA) probes as detection tools, were used for detection and validation of marker genes. The detailed procedures were described previously [40].

### 4.5. Statistical Analysis

Gene expression from qPCR data was statistically analyzed by two-tailed Student’s *t*-test, a Kruskal-Wallis test, and Receiver Operating Characteristic curves analysis. The efficacy of each marker gene was also compared with that of CEA, the current tumor marker for colorectal cancer. All statistical analyses were conducted using the SPSS statistical software program (version 15.0) (SPSS, Inc., Chicago, IL, USA).

## 5. Conclusions

In this study, we are the first group to evaluate the early diagnostic role of ECT2 in circulating colorectal cancer cells in the peripheral blood. We observed that the expression of ECT2 can therefore serve as an alternative measurement that can compensate for the inadequacy of the current CEA test in colorectal cancer patients.

## Figures and Tables

**Table 1:** Characteristics of colorectal patients and healthy controls.

	Patients (*n* = 90)		Control (*n* = 151)	
Characteristic	No.	%	No.	%
Age, year				
Mean	66.45		36.29	
Range	36–90		22–69	
Gender				
Female	45	50	101	67
Male	45	50	50	33
Primary Tumor				
Colon	59	61	N/A	
Rectal	33	34		
Rectosigmoid	3	3		
Others	2	2		
Stage				
I	6	6	N/A	
II	11	12		
III	30	33		
IV	43	47		
Differentiation				
Well	2	2	N/A	
Moderate	81	90		
Poor	5	6		
Unspecified	2	2		
CEA, ng/mL				
≥5	46	51	N/A	
<5	44	49		

N/A: not applicable; CEA: carcinoembryonic antigen.

**Table 2:** Proportion of elevated ECT2 and CEA detected in serum in different combination of patients with colorectal cancer.

CEA/ECT Expression	Stage I and II, *n* = 17 (%)	Stage III, *n* = 30 (%)	Stage IV, *n* = 43 (%)	All, *n* = 90 (%)
CEA^+^ECT2^−^	4 (24)	9 (30)	9 (21)	22 (24)
CEA^−^ECT2^+^	9 (53)	11 (37)	10 (23)	30 (33)
CEA^+^ECT2^+^	1 (6)	6 (20)	6 (20)	24 (27)
CEA^+^ and/or ECT2^+^	14 (82)	26 (87)	26 (84)	76 (84)
CEA^−^ECT2^−^	3 (18)	4 (13)	7 (16)	14 (16)

Cut-off level for CEA is 5 ng/mL and for ECT2 is 1.79 ng/mL.

## Supplementary Materials

Supplementary materials can be found at www.mdpi.com/1422-0067/18/4/743/s1.

## Abbreviations

CTCs	Circulating tumor cells
ECT2	Epithelial cell transforming sequence 2 oncogene
AUC	Area under the curve
CEA	Carcinoembryonic antigen
EMT	Epithelial to mesenchymal transition
qPCR	Quantitative PCR
KW	Kruskal-Wallis
