# Supplementary material for: Early Assessment of Colorectal Cancer by Quantifying Circulating Tumor Cells in Peripheral Blood: ECT2 in Diagnosis of Colorectal Cancer
Source: Int J Mol Sci. 2017 Mar 31;18(4):743. doi: 10.3390/ijms18040743 (PMC5412328; doi:10.3390/ijms18040743)
Supplement: Supplementary file 1 [file ijms-18-00743-s001.pdf]

Supplementary Materials: Early Assessment of Colorectal Cancer by Quantifying Circulating Tumor Cells in Peripheral Blood: ECT2 in Diagnosis of Colorectal Cancer

Chih-Jung Chen, Wen-Wei Sung, Hung-Chang Chen, Yi-Jye Chern, Hui-Ting Hsu, Yueh-Min Lin, Shu-Hui Lin, Konan Peck, Kun-Tu Yeh

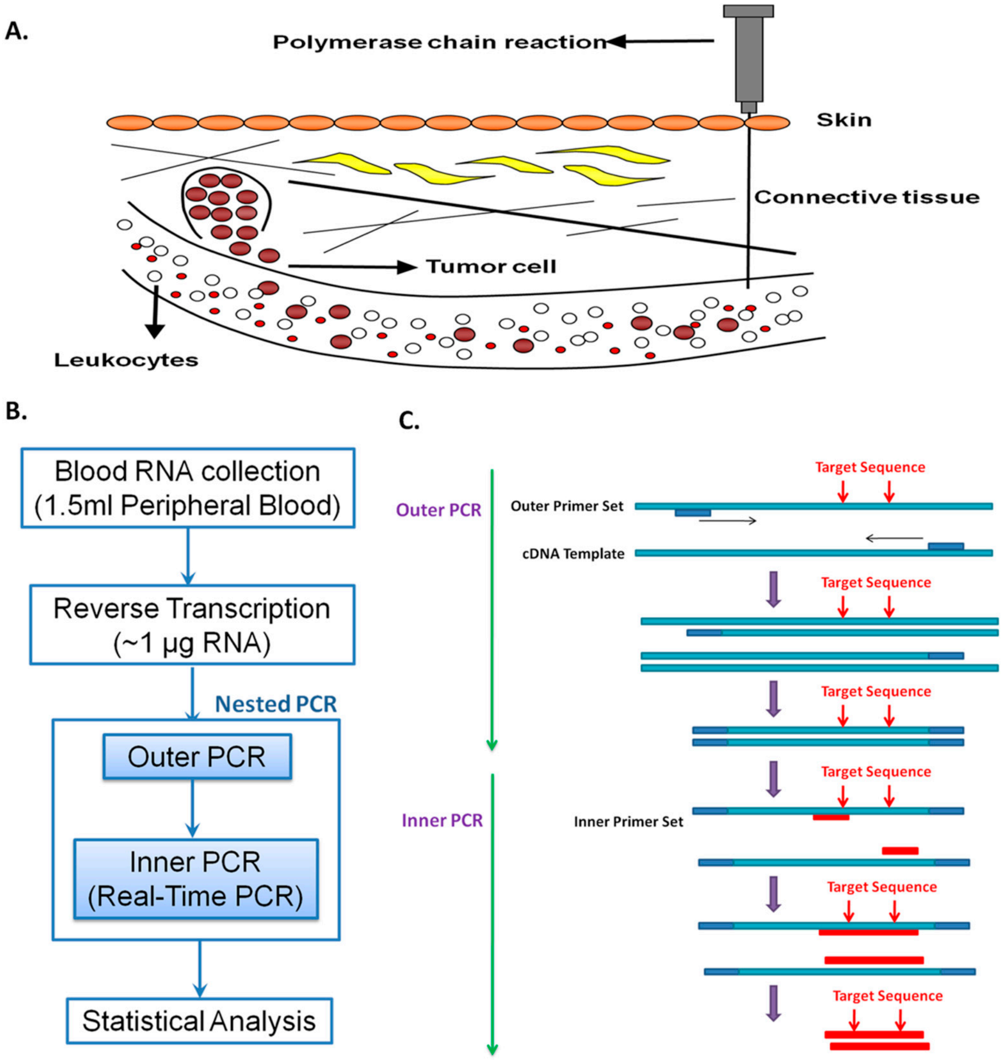

Figure S1. Candidate marker genes examined for detection of circulating tumor cells by nested real-time polymerase chain reaction (PCR). (**A**) Tumor cells undergoing the epithelial to mesenchymal transition (EMT) acquire motile propensities and enter the blood. A 1.5 mL sample of peripheral blood from colorectal cancer patients was directly taken for candidate marker gene determination. (**B**) RNA was extracted from cells isolated from the peripheral blood. After reverse transcription of RNA, expression of candidate marker genes was quantified by nested real-time PCR. (**C**) Nested real-time PCR diagram. First, a target sequence is amplified by an outer primer set with a few rounds. The PCR products from the first PCR reaction are subjected to real-time PCR using a hydrolysable fluorescent probe for detection. Two rounds of PCR as well as sequence-based probe detection ensure the sensitivity and specificity of this method.
